# Big data tax collection and management, enterprise financialization and enterprise innovation: Quasi-natural test evidence based on the "Golden Tax Phase III"

**DOI:** 10.1371/journal.pone.0315222

**Published:** 2024-12-19

**Authors:** Deliang Zhou, Zexuan Zhang

**Affiliations:** School of Accounting, Lanzhou University of Finance and Economics, Lanzhou, People’s Republic of China; Kathmandu Model College, Tribhuvan University, NEPAL

## Abstract

In the era of digital economy, big data tax collection and management has become an important governance tool for digital government. In this study, the quasi-natural test environment provided by the "Golden Tax Phase III" policy launched in 2013 and the method of propensity score matching and differentiation (PSM-DID) were used on combination of the samples of A-share listed enterprises in Shanghai and Shenzhen during 2010–2021 to analyze and demonstrate the impact of this policy on the innovation of the listed enterprises. To ensure the robustness of these findings, various statistical techniques such as parallel trend tests, placebo tests, and the explained variable replacement were employed. Additionally, an influence mechanism test was conducted to examine the mediating effect of big data tax collection and management on enterprise innovation, revealing the reduction of enterprise financialization. Furthermore, moderating effect tests and heterogeneity analyses were also performed, and the results showed that the agency costs and financing constraints play a negative role in regulation, and the promotion effect of big data tax collection and management on enterprise innovation is more significant in enterprises with high information transparency and non-high-tech enterprises. Finally, in the further study and economic consequence test, it is found that big data tax collection and management can promote the high-quality development of enterprises while promoting enterprise innovation. The conclusions of this study are helpful for government departments to continuously promote big data tax collection and management, promote the implementation of innovation-driven strategic policies, and promote high-quality economic development.

## 1. Introduction

In the current context of globalization, all countries are committed to promoting the transformation and development of economic digitalization, and strive to complete the strategic layout of the digital economy [1]. China has clearly stated that data is one of the important production factors, indicating that China regards data-driven high-quality economic development as its core strategic direction, so as to improve the modernization level of national governance [2]. Among them, taxation plays a basic, pillar and guarantee role in national governance, and promoting big data tax collection and management to be significant for realizing the modernization of the national governance system and governance capacity [3]. In June 2022, the Central Committee of the Communist Party of China and the State Council issued the *Guiding Opinions on Strengthening the Construction of Digital Government*, proposing to comprehensively promote the digital transformation of government affairs, promote digital governance, and realize the modernization of government governance capabilities [4]. Additionally, in October 2022, the report of the 20^th^ National Congress of the Communist Party of China proposed to accelerate the construction of a cyber power and a digital power, pointing out the direction for China to further build a digital government and achieve high-quality development of the digital economy [5]. In recent years, with the extensive application of Internet big data technology and the firm support of Chinese Government for the reform of the tax collection and management system, especially the successful completion of the "Golden Tax Phase III" project, the tax collection and management model of China has ushered in unprecedented changes. Overall, these changes indicate that the tax collection and management of China has truly realized informatization and entered a fresh new era [6].

As the primary driving force for development, scientific and technological innovation plays a pivotal role in the process of building a modern economic system, and it is of great significance to lead the new transformation of Chinese production modes and explore the new momentum of the economic growth of China. As a result, the Government is increasingly focusing on developing appropriate policies to incentivize enterprise innovation. Specifically, tax policy is considered as an effective means for the Government to influence enterprise behaviors and innovation activities [7–9]. Based on this, it is of great theoretical value and practical significance to deeply understand the role of big data tax collection and management, and explore the impact of big data tax collection and management on enterprise innovation and its internal mechanism, which is of great theoretical value and practical significance for the long-term development of enterprises in the new era.

From the perspective of the existing literatures, scholars mainly discuss on the influencing factors of enterprise innovation from the aspects of tax enforcement, environmental information disclosure, enterprise financialization and stock price characteristics. For example, stricter tax enforcement can promote internal enterprise innovation, and its mechanism analysis suggests that the enhancement of enterprise innovation is driven by improved governance level, that is, stricter tax enforcement restraint managers’ bribery and irregularities [9]. Environmental information disclosure significantly promotes enterprise innovation and promotes exploratory innovation more than exploratory innovation, and media attention has an "inverted U-shaped" moderating effect on the relationship between environmental information disclosure and enterprise innovation [10]. According to the studies of Zhang, et al (2021), the allocation of financial assets by entities does not necessarily achieve the optimal allocation of resources, and even crowds out the innovation investment of enterprises to a certain extent and ultimately reduces the innovation quality of entities [11]. There is a significant difference in the marginal effect of financial asset allocation on innovation quality among physical enterprises for the purpose of preventing reserve motivation or capital arbitrage motivation. The inhibitory effect of financialization behaviors on innovation quality of entities will weaken as the degree of financing constraints decreases. The study of Anqi Wang (2003) pointed out that, stock price trait information can optimize the efficiency of enterprise resource allocation, alleviate financing constraints, and promote enterprise innovation [12].

Scholars mainly discuss the impact of big data on tax collection and management in the existing literature, such as its impact on enterprise financialization, corporate financing constraints, and enterprise total factor productivity. For example, big data tax collection and management can significantly promote the technological innovation behavior of enterprises [13]. The impact of the "Golden Tax Phase III" project on the informatization of tax collection and management and financing constraints from the perspective of earnings quality, revealing the effect of tax collection and management informatization on improving the quality of corporate earnings [14]. In addition, the digital upgrade of tax collection and management has a positive impact on the total factor productivity of enterprises and promotes the acceleration of enterprise innovation through the quasi-natural experiment of the "Golden Tax Phase III" [15].

In view of the lack of existing research on the impact of big data tax collection and administration on enterprise innovation, this paper takes Shanghai and Shenzhen A-share listed companies from 2010 to 2021 as a sample, and analyzes and demonstrates the impact of this policy impact on the innovation of listed companies with the help of the exogenous impact of the "Golden Tax Phase III" project pilot. The results show that big data tax collection and management has a significant role in promoting the innovation of listed enterprises.

Compared with the previous literatures, the contribution margin of this paper includes: Firstly, starting from big data tax collection and management, the impact of big data tax collection and management on enterprise innovation is explored in this study, enriching relevant study on the influencing factors of enterprise innovation. Secondly, it reveals the inherent mechanism of big data tax collection and management on enterprise innovation. This paper is based on data from the listed enterprises from 2010 to 2021, empirically testing the impact of big data tax collection and management on enterprise innovation, and further exploring the mediating effect of enterprise financialization in big data tax collection and management and enterprise innovation, as well as the moderating effect of agency costs and financing constraints, thus revealing the inherent mechanism of the impact of big data tax collection and management on enterprise innovation. Thirdly, robustness test, heterogeneity analysis, and economic consequence test are further conducted in this paper, which can not only provide theoretical inspiration and methodological reference for subsequent related study in this field, but also provide useful policy insights for promoting enterprise innovation using the big data tax collection and management.

The upcoming sections of this essay are structured as follows: Part II focuses on literature review and study assumption, while Part III describes the study design. Part IV presents the research methods, and Part V conducts the further study. Part VI describes the economic consequences. Finally, Part VII concludes the entire study.

## 2. Literature review and study assumption

### 2.1 Literature review

In the current digital era, the wide application of big data technology has profoundly changed the management and operation mode of various industries, also in the field of tax collection and management. Through the implementation of the "Golden Tax Phase III" project, tax collection and administration in China has achieved digital upgrading, using big data technology to carry out informatization, networking and intelligent management. Big data tax collection and management can deeply mine and analyze massive taxpayer data, achieve more efficient and accurate tax collection management and promote the standardization and facilitation of tax collection and collection. The comprehensive application of big data technology has had a profound impact on the improvement of enterprise innovation capabilities. Some scholars believe that there is a positive impact between big data tax administration, corporate financialization, and corporate innovation. Zhang et al. (2023) studied the relationship between big data tax collection and management and stock price synchronization through quasi-natural test evidence based on the "Golden Tax Phase III" project, which is conducive to improving the response speed of stock prices and the effectiveness of market resource allocation, thereby promoting the innovation activities of enterprise [16]. Ji et al. (2023) studied whether big data tax collection and management can the illegal behaviors of enterprises, and big data tax collection and management can promote integrity behaviors and standardized operations of enterprises, which is conducive to the sustainable development of enterprise innovation [5]. Liu et al (2022) studied the impact of informatization of tax collection and management on the uncertainty of enterprise taxation, and the implementation of digital tax collection and management has improved the efficiency and accuracy of tax collection and management, reduced the tax uncertainty caused by the adjustment of tax policy for enterprises, and enabled enterprises to more accurately predict and plan the tax burden, reduce the risk of business decision-making, and improve the enterprises innovation stability and ability [17].

However, there are still some controversies and issues that need further research on the positive effects of big data tax collection and management on enterprise financialization and enterprise innovation. Some studies have pointed out that although big data tax administration provides more information and support, it does not necessarily directly promote corporate financialization and corporate innovation. Niu et al (2023) studied the impact of digital upgrading of tax collection and management on the stickiness of enterprise expenses through a quasi-natural test of the "Golden Tax Phase III" project, and found that big data tax collection and management may have a certain inhibitory effect on the enterprise innovation ability [18]. Yang et al (2023) studied on the impact of tax collection and management on the cash holding of enterprises, and the digital upgrade of tax collection and management may have certain constraints on the innovation ability of enterprises [19].

### 2.2 Study assumption

From the perspective of the implementation of the "Golden Tax Phase III" policy, this paper examines how big data tax collection and management can promote enterprise innovation by influencing the financialization of enterprises.

This policy through the implementation of tax collection and management information reform, the use of advanced information technology, the implementation of the whole process of electronic tax-related management, improve the tax administrative law enforcement network system, strengthen the tax administrative law enforcement supervision ability, thereby enhancing the reliability and controllability of enterprise tax-related management [20]. The implementation of "Golden Tax Phase III" not only improves the quality of tax-related administrative law enforcement, but also enhances the sense of tax-related security of enterprises. In addition, the third phase of the Golden Tax also vigorously promotes the reduction of the tax burden of small and micro enterprises, supports enterprises to carry out scientific and technological innovation, and improves the convenience and efficiency of enterprise tax management. These measures jointly promote the innovation activities of enterprises and provide strong support for the technological innovation and development of enterprises. The existing research finds that after the pilot implementation of the third phase of the Golden Tax Project, enterprises have increased their investment in innovation, which is mainly obtained through the corporate governance effect and information quality effect of big data tax collection and management, and the innovation output of enterprises has been significantly improved [21].

However, there are also some drawbacks in the implementation of this policy, and through the reform of the tax inspection system, the income tax burden rate of enterprises has been increased, and the tax burden of enterprises has been increased. This increased tax burden could lead to financial pressure on companies, which in turn limited their investment in innovation activities such as R&D, technology upgrading, and talent development.

In order to further study and verify the impact mechanism of big data tax collection and management on enterprise innovation, and to provide more accurate and comprehensive policy and technical support, the digital upgrade of tax collection and administration can help enterprises better cope with market challenges and competitive pressures, and promote the technological progress and innovation ability of enterprises, the following research hypotheses are proposed:

H1: Big data tax collection and management has a promoting impact on the innovation ability of enterprises.

In order to further study and verify the mediating role of enterprise financialization in big data tax collection and management and enterprise innovation, the following research hypotheses are proposed:H2: Enterprise financialization plays an intermediary role between big data tax collection and management and enterprise innovation.

## 3. Study design

### 3.1 Sample selection and data sources

In 2013, the Golden Tax Phase III project was implemented in Shanxi, Shandong (except Qingdao) and Chongqing in China, launched in Guangdong (except Shenzhen), Inner Mongolia and Henan in 2014, promoted and applied in Hebei and other places in 2015, and was put into operation in 16 provinces and cities such as Shanghai and Beijing in 2016, thus achieving full coverage of Chinese mainland. In order to avoid the interference of the time span before and after the implementation of the policy, the study sample used in this paper is the relevant data of A-share listed enterprises during 2010–2021, and the following data processing is performed on the initial sample: (1) Due to the significant differences between the financial statement structure and data of the financial industry and other industries, the financial industry sample was excluded; (2) In view of the large gap between the financial status and operating conditions of ST companies and those of normal operating companies, in order to avoid the impact of industry particularity on the analysis results, the ST sample was excluded; (3) The missing samples of the required variables are eliminated, (4) The influence of extreme values on the results are eliminated, (5) The required variables are shrunk, 25,251 annual-company data had been obtained, the data sourced from CSMAR Database, and STATA17.0 is used for statistical analysis in this paper.

### 3.2 Definition of variables and model specification

#### 3.2.1 Definition of variables

Big data tax collection and management. The launch of the "Golden Tax Phase III" is used in this to measure whether the big data tax collection and management (Post) has been implemented. It should be noted that the launch of the "Golden Tax Phase III" should be promoted through a certain period, thus, according to the method of Kai, et al (2021), if it is launched in the region in the first half of the year, big data tax collection and management will be deemed to have been implemented this year, and if it is launched in the second half of the year, big data tax collection and management will be deemed to have been implemented in the next year [22].

Financialization of businesses. According to the opinions of Chengsi Zhang (2019), the increasing proportion of financial investment is regarded as the key manifestation of the financialization of micro enterprises, the proportion of enterprise financial assets in total assets is used in this paper to describe the financialization level of real enterprises [23].

Enterprise innovation. Following the thoughts of Liu et al (2020), the total number of applications for invention patents, utility models and design patents plus the natural logarithm of 1/ln (1 + R&D expenditure) is used to measure enterprise innovation. In order to eliminate the problem caused by the gap between R&D results and practical application on the measurement of enterprise innovation quality, the total number of applications for invention patents, utility model patents and design patents is added to the natural logarithm of 1, and the weight of the three patents is calculated according to the value of 3:2:1/ln (1 + R&D expenditure) as a robustness test variable [24].

Control variables. By referring to the literatures, a series of control variables were selected with reference to the existing literature, including asset-liability ratio (Lev), total assets net profit rate (ROA), return on equity (ROE), gross profit rate (Gross Profit), net profit margin on sales (Net Profit), current ratio (Liquid), quick ratio (Quick), cash flow ratio (Cashflow) and tangible assets ratio (Tangible). Table 1 contains the definitions of the main variables used in this study.

**Table 1:** Variable description.

Variable type	Variable name	Variable symbols	Variable interpretation
Explained variable	Enterprise innovation	InnoEff1	Total number of applications for invention patents, utility models and design patents plus the natural logarithm of 1/ln (1 + R & D expenditure)
Interpretation variable	Big data collection and management	Post	In the current year and subsequent years of the "Golden Tax Phase III" carried out by the region, the value is 1, otherwise it is 0
Intermediary variable	Enterprise financialization	FINRATIO	The ratio of enterprise financial assets to total assets
Control variables	Enterprise scale	Size	The natural logarithm of annual total assets
	Asset-liability ratio	Lev	Total liabilities at the end of the year/total assets at the end of the year
	Return on assets	ROA	Net profit/average balance of total assets
	Return on equity	ROE	Net profit/ average balance of shareholder equity
	Gross profit rate	GrossProfit	(Operating revenue- operating costs)/ Operating revenue

#### 3.2.2 Study model

Since its launch in 2013, the "Golden Tax Phase III" system has been gradually promoted nationwide, and it has been implemented in batches and regions through four years. The pilot implementation of the policy in batches has a quasi-natural test nature. Since this policy was implemented in different provinces in 2013, 2015, 2016 and other years, this paper uses the "Golden Tax Phase III" to conduct quasi-natural experiments, and a multi-period difference-in-difference model is used to study the impact of big data tax collection and management on enterprise innovation, and the Model (1) is constructed as follows:

$$InnoEff1=\alpha_{0}+\alpha_{1}{Post}_{pt}+{\gamma X}_{it}+\phi_{p}\times \theta_{t}+\phi_{p}+\varepsilon_{it}$$ (1)

In Model (1), i, p, and t represent enterprises, provinces, and years; Respectively, enterprise innovation is represented by *InnoEff1*, and control variables are represented by *X*_*it*_; *φ*_p_ and*φ*_p_ ×*φ*_t_ are represented by A and B for the provincial fixed effect, the provincial fixed effect × the annual fixed effect, respectively; *ε*_*it*_ represents residual terms.

To test the relationship between big data tax collection and management, enterprise financialization and enterprise innovation, Model (2) is set as follows:

$$InnoEff1=\alpha_{0}+\alpha_{1}{Post}_{pt}+\alpha_{2}FINRATIO+{\gamma X}_{it}+\phi_{p}+\phi_{p}\times \theta_{t}+\varepsilon_{it}$$ (2)

### 3.3 Descriptive statistics

S1 Table presents the descriptive statistics of the main variables in this study. The mean of InnoEff1 is 0.16, indicating an average increase of 16% in innovation efficiency. The mean of Post is 0.68, indicating that 68% of enterprises in the sample have implemented policies. The mean value of FINRATIO is 0.04, and 4% of enterprises in the surface sample have achieved enterprise financialization. The other control variables are basically consistent with existing study.

## 4. Research methods

### 4.1 Basic regression results

In this study, stepwise regression analysis is used to test hypothesis H1, and Table 2 summarizes the study results. Column (1) shows the results with no fixed effect, column (2) shows only corporate control, and column (3) shows the situation for 300 holding companies and provinces. The results show that the coefficient of Post is 0.030, and there is a significant positive correlation at the level of 1%. This indicates that big data tax administration has a promoting effect on the innovation efficiency of enterprises, consistent with hypothesis H1.

**Table 2:** Basic regression.

	(1)	(2)	(3)
VARIABLES	OLS	FE	FE
Post	0.014***	0.014***	0.014***
	(12.35)	(10.42)	(10.54)
Size	0.019***	0.023***	0.022***
	(39.93)	(16.16)	(16.19)
Lev	-0.003	-0.012**	-0.012**
	(-0.85)	(-2.10)	(-2.10)
ROA	0.018	-0.073***	-0.071***
	(0.88)	(-3.58)	(-3.48)
ROE	0.034***	0.025***	0.024***
	(3.52)	(2.76)	(2.68)
GrossProfit	-0.024***	0.038***	0.038***
	(-7.02)	(4.72)	(4.74)
Constant	-0.274***	-0.356***	-0.321***
	(-27.24)	(-11.95)	(-9.86)
Observations	25,251	25,251	25,251
Adj.R^2^	0.106	0.102	0.105
Number of id		3,888	3,888
id FE		YES	YES
Province FE			YES

t-statistics in parentheses

*** p<0.01

** p<0.05

* p<0.1

### 4.2 Robustness test

#### 4.2.1 Test for parallel trends

Before using the difference-in-difference model to test the effect of policy evaluation, the basic assumption of the assumption is that the equilibrium trend hypothesis needs to be satisfied. It is required that the difference between the test group affected by the launch of the "Golden Tax Phase III" system and the control group not affected by the launch of the "Golden Tax Phase III" system is after the implementation of the "Golden Tax Phase III" system, and the change trend of enterprise innovation indicators between the control group and the test group before the implementation of the "Golden Tax Phase III" system should be consistent. In this study, 2013 is used as the base year to generate dummy variables (pre_2, pre_1, current, post1, post 2, post 3). pre_2 and pre_1 refer to the two and one years before the policy implementation, while current, post_1, post_2, and post_3 represent the year of implementation and the subsequent years. The regression results in Table 3 show that the coefficients of pre_2 and pre_1 are significant at the 10% level, suggesting a similar trend of change between the test and control groups before the policy’s implementation. When the period before the policy is excluded as the base year, the parallel trend hypothesis is satisfied. The coefficients of post_1, post_2 and post_3 are all significantly negative at the 1% level, indicating that the "Golden Tax Phase III" has a significant moderating effect on the enterprise innovation.

**Table 3:** Test for parallel trends.

	(1)
VARIABLES	Parallel trend
pre_3	0.001
	(0.32)
pre_2	0.001
	(0.60)
current	0.001
	(0.56)
post_1	0.004***
	(3.15)
post_2	0.011***
	(3.22)
Observations	27,411
Adj.R^2^	0.069

Robust t-statistics in parentheses

*** p<0.01

** p<0.05

* p<0.1

**Fig 1:**
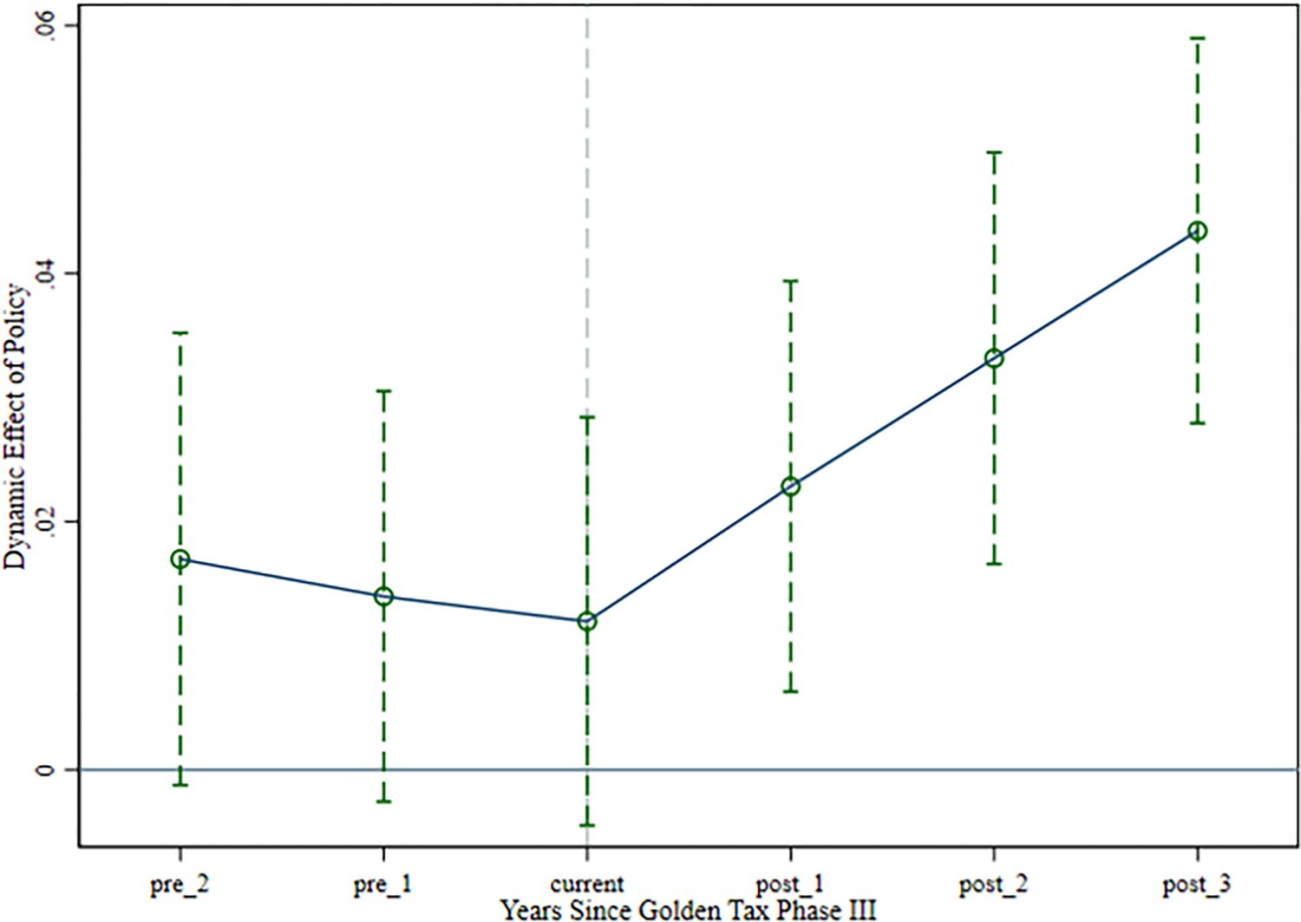
Plot of parallel trend test for enterprise innovation.

The results of the parallel trend test show that the conclusions of this research satisfy the hypotheses related to the parallel trend test. We also plotted parallel trends in enterprise innovation. Details are shown in Fig 1 below.

#### 4.2.2 Placebo test

**Fig 2:**
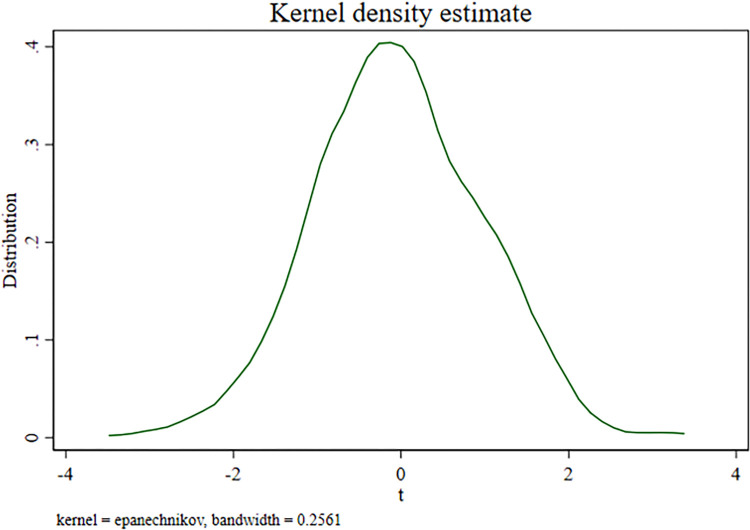
Placebo test.

The benchmark regression results in this paper may be a coincidence, due to chance or random factors. For example, with the passage of time, the innovation efficiency of enterprises gradually increases, and the benchmark regression results may not have any correlation with the implementation of the "Golden Tax Phase III". To dispel this concern, counterfactual estimation is made in this paper according to Chettyetal (2009). Specifically, the pilot area and pilot year of the Golden Tax Phase III project were randomly constructed, that is, the big data tax collection and management (Post) was randomly assigned, and this process was repeated 500 times to make counterfactual estimates, and the results are shown in Fig 2. The t-value of the estimation coefficient of big data tax collection and management (Post) is concentrated around 0, indicating that the estimation coefficient of Post is not significant. This means that the above concerns do not exist, and the benchmark regression results do reflect the impact of the implementation of the "Golden Tax Phase III", rather than a coincidence of chance [25].

#### 4.2.3 PSM-DID

Due to the systematic differences between samples that may affect the study results, to address this issue, the propensity score matching method (PSM) of 1:1 nearest neighbor matching for testing is used in this paper. The logit model is used to estimate the propensity score value, and then the propensity score is used for one-on-one nearest neighbor matching. Finally, the samples matched with propensity scores were used for regression.

As shown in Table 4, the given regression results indicate that after PSM matching, the coefficient of the interaction item_diff is negative, -0.019, which is statistically significant at the 1% level. This indicates that the conclusion still holds after eliminating the inherent differences between the control group and the treatment group.

**Table 4:** PSM-DID test.

	(1)
VARIABLES	PSM-DID
time	0.036***
	(12.66)
treated	-0.000
	(-0.10)
_diff	-0.019***
	(-5.08)
controls	control
Constant	0.140***
	(55.93)
Observations	14,974
Adj.R^2^	0.020

t-statistics in parentheses

*** p<0.01, ** p<0.05, * p<0.1

#### 4.2.4 Replace the explained variable

In this study, the robustness test is carried out by replacing the measurement method of the explanatory variable enterprise innovation efficiency, so as to reduce the impact of the error of the measurement method on the study results. By referring to the method of Liu et al. (2020), another method is used to measure enterprise innovation, the OLS model is used to regress for hypothesis H1, and the impact of big data tax collection and management on enterprise innovation efficiency is reported in S2 Table. Column (1) shows the results with no fixed effect, with the coefficient of Post is 0.032 and is significantly positively correlated at the 1% level. Column (2) shows the situation where only the controlling enterprise is considered, with the coefficient of Post is 0.031 and is significantly positively correlated at the 1% level. Column (3) shows the situation of the controlled enterprises and provinces, with the coefficient of Post is 0.031 and is significantly positively correlated at the 1% level. The results in S2 Table are consistent with the benchmark regression, further proving the validity of this conclusion, and the tax collection and management of big data has a promoting effect on the innovation efficiency of enterprises.

### 4.3 Influence mechanism test

#### 4.3.1 Intermediary mechanism test

Based on the above theoretical analysis, it can be seen that one of the important influencing mechanisms is that after the implementation of the "Golden Tax Phase III" project, big data tax collection and management greatly reduces the financialization of enterprises, and the financialization of enterprises inhibits enterprise innovation, resulting in big data tax collection and management promoting enterprise innovation. In this paper, the Sobel-Goodman mediation effect test is used to test whether the transmission mechanism of enterprise financialization is established, and the results of the mediating effect test with enterprise financialization as the mediating variable are shown in column (1) of Table 5. The coefficient of enterprise financialization (FINRATIO) is 0.06, which is significantly negative at the 1% level, and the coefficient of big data tax collection and administration (Post) is 0.031, which is significantly positive at the 1% level. According to the regression results, the intermediary effect test of enterprise financialization is passed, which verifies the basic hypothesis of this paper. After the launch of the "Golden Tax Phase III", Big data tax collection and management can promote the efficiency of enterprise innovation by reducing the financialization of enterprises.

**Table 5:** Influence mechanism test.

	(1)	(2)	(3)
VARIABLES	FINRATIO	Agency costs	Financing constraints
FINRATIO	-0.060***		
	(-6.40)		
Post	0.031***	0.029***	0.040***
	(23.82)	(21.72)	(15.28)
DL01		-0.022**	
		(-2.46)	
FC			-0.043***
			(-9.89)
Lev	0.034***	0.036***	-0.018***
	(7.71)	(7.70)	(-3.19)
ROA	0.058**	0.051	0.065**
	(1.96)	(1.58)	(2.04)
ROE	0.082***	0.086***	0.076***
	(6.47)	(6.13)	(5.46)
GrossProfit	-0.021***	-0.008	-0.018***
	(-4.80)	(-1.09)	(-3.91)
NetProfit	-0.025***	-0.036***	-0.035***
	(-3.62)	(-4.55)	(-4.72)
Liquid	-0.005***	-0.006***	-0.005***
	(-3.53)	(-3.76)	(-2.96)
Quick	0.004**	0.005***	0.003*
	(2.53)	(2.80)	(1.89)
Cashflow	-0.013	-0.019*	-0.052***
	(-1.22)	(-1.73)	(-4.74)
Tangible	0.040***	0.037***	0.043***
	(5.78)	(5.24)	(6.11)
Constant	0.102***	0.105***	0.144***
	(15.12)	(15.08)	(19.13)
Observations	17,330	16,204	16,204
Adj.R^2^	0.068	0.067	0.089

t-statistics in parentheses

*** p<0.01

** p<0.05

* p<0.1

#### 4.3.2 Moderating effect test

On the basis of the results of main effect analysis and intermediary effect analysis, in this paper also considers the influence of internal factors of enterprises, further analyzes and tests the moderating role of internal factors in the relationship between big data tax collection and management and enterprise innovation, and selects agency costs and financing constraints with reference to previous literature.

*(1) Agency costs*. The strengthening of big data tax collection and management is equivalent to a strong and effective external governance mechanism, which can realize the joint supervision of the management by the tax department and shareholders, and then produce a synergistic effect. With the support of the Internet and big data, the tax collection and management department can grasp the operation status of the enterprise in real time, and realize the supervision of the operation status of the enterprise, prevent the occurrence of the management from encroaching on the interests of major shareholders, and then reduce the problem of entrustment and agency between shareholders and managers, alleviate agency conflicts, and improve the innovation efficiency of enterprises. In this paper, the moderating effect test method is used to test whether the agency cost transmission mechanism is valid, according to the method of Tong Yong (2021), the agency cost (DL01) is determined as a moderating variable, and it is decentralized [26]. Column (2) of Table 5 presents the results of the regression with agency cost as the moderating variable. The coefficient of the interaction between Post and DL01 is 0.053, which is significantly negative at the level of 1%, indicating that agency cost plays a moderating role in big data tax collection and enterprise innovation.

*(2) Financing constraints*. Big data tax collection and management can alleviate the problem of information asymmetry between external investors and enterprises, gain the trust of investors and avoid adverse selection, thereby reducing the difficulty of enterprises to obtain funds externally. The possibility of adverse selection, thereby greatly improving market transparency and effectively easing financing constraints. On the other hand, the "innovation promotion effect" and "economies of scale effect" of financing constraint alleviation have a significant effect on enterprise innovation: in terms of "innovation promotion effect", the effective alleviation of enterprise financing constraints provides necessary and sufficient funding sources for enterprises to innovate, which can promote enterprises to increase innovation efforts [27]. According to the measurement method of Jun and Zheng (2020) et al., this paper uses the FC index to measure the degree of enterprise financing constraints, and the larger the FC index value, the greater the financing constraints [28]. Column (3) of Table 5 presents the results of the regression with financing constraints as the moderating variable. The coefficient of the interaction term between Post and FC is 0.025, which is significantly negative at the level of 1%, indicating that financing constraints play a moderating role in big data tax collection and enterprise innovation.

### 4.4 Heterogeneity test

#### 4.4.1 Company information transparency

Analysts conducted study and analysis on the financial status of listed enterprises, and the resulting analysis report will promote the disclosure of enterprise characteristics information in the stock market [29], which will improve the overall information transparency of the capital market. Analysts have more access to information and professional analysis and forecasting capabilities, and can transmit more abundant information about enterprise characteristics to the capital market [30]. This paper uses the number of enterprise analyst trackers to measure the information environment of the capital market, that is, the more analyst trackers, the higher the information transparency of the company. According to the median number of analyst trackers, the samples were divided into a lower group and a higher group with a higher number of analyst trackers, and the regression results of heterogeneity analysis are shown in Table 6. Column (1) is the group with a higher number of analyst tracks, and the coefficient of Post is 0.03, which is significantly positive at the 1% level. Column (2) is the group with a low number of analyst trackers, and the coefficient of Post is 0.02, which is significantly positive at the 1% level. This shows that compared with enterprises with higher information transparency, big data tax collection and management has a more significant effect on promoting enterprise innovation.

**Table 6:** Heterogeneity test.

	(1)	(2)	(3)	(4)
VARIABLES	High	Low	High-tech	Non-high-tech
Post	0.030***	0.020***	0.026***	0.038***
	(22.11)	(2.89)	(22.52)	(18.38)
Lev	0.019***	0.018	0.023***	0.011
	(2.67)	(0.61)	(3.54)	(0.94)
ROA	-0.197***	-0.242***	-0.144***	0.007
	(-5.24)	(-2.99)	(-4.87)	(0.13)
ROE	0.089***	0.058*	0.044***	0.019
	(5.57)	(1.96)	(3.69)	(0.89)
GrossProfit	0.040***	0.042	0.047***	0.010
	(3.86)	(1.22)	(5.31)	(0.56)
NetProfit	0.003	0.023	0.013*	-0.014
	(0.25)	(1.42)	(1.92)	(-1.05)
Liquid	-0.000	0.003	0.001	0.001
	(-0.18)	(0.29)	(0.28)	(0.31)
Quick	0.000	-0.005	-0.001	-0.002
	(0.14)	(-0.50)	(-0.39)	(-0.46)
Cashflow	0.032***	-0.104***	0.009	-0.016
	(3.04)	(-2.66)	(0.92)	(-0.93)
Tangible	-0.057***	-0.063*	-0.076***	-0.047***
	(-6.25)	(-1.80)	(-8.80)	(-2.69)
Constant	0.206***	-0.116	0.028	0.066
	(3.91)	(-0.92)	(0.48)	(0.81)
Observations	12,147	1,849	11,659	5,671
Adj.R^2^	0.731	0.805	0.731	0.699
id FE	YES	YES	YES	YES
Province FE	YES	YES	YES	YES

Robust t-statistics in parentheses

*** p<0.01

** p<0.05

* p<0.1

#### 4.4.2 Scientific and technological content

In order to verify whether the impact of the launch of the "Golden Tax Phase III" on the innovation activities of enterprises varies according to the high-tech industry, based on the study of Li et al. (2016) according to the GB/T 4754 industry classification standard of the National Bureau of Statistics of China, the samples of industries such as office machinery are classified as high-tech enterprises, and the rest are non-high-tech enterprises [31]. High-tech enterprises and non-high-tech enterprises were grouped for regression, and the regression results are shown in Table 6. Column (3) is a high-tech enterprise, and the Post coefficient is 0.026, which is significant at the level of 1%. Column (4) is a non-high-tech enterprise, and the Post coefficient is 0.038, which is significant at the level of 1%. It shows that the positive impact of the launch of the "Golden Tax Phase III" project on enterprise innovation is more obvious in non-high-tech industries. The reason for the above results may be that the high-tech industry is a knowledge- and technology-intensive industry, and the technology has developed relatively maturely, and there is some slackness in innovation. Non-high-tech industries such as agriculture, forestry, animal husbandry, fishery, etc., still have a lot of room for improvement in innovation and study and development, which in turn affects their innovation investment level.

## 5. Further study

In this paper, the explanatory variable enterprise innovation is divided into ambidextrous innovation, which is divided into development innovation and exploratory innovation [32]. Columns (1) and (2) in Table 7 are the regression results of big data tax collection and management on development innovation and exploratory innovation, respectively. Column (1) shows that Post’s regression coefficient is insignificant and negative. Column (2) shows a coefficient of 0.006 for Post, which is significantly positive at the 1% level. The results show that there are differences in the impact of big data tax collection and administration on ambidextrous innovation, which is that exploratory innovation is significantly promoted, but there is no significant impact on development innovation.

**Table 7:** Dual innovation.

	(1)	(2)
VARIABLES	Development innovation	Exploratory innovation
Post	-0.000	0.006***
	(-1.23)	(10.76)
Lev	-0.001	-0.024***
	(-0.32)	(-5.85)
ROA	-0.028***	-0.079***
	(-3.25)	(-4.13)
ROE	0.006*	0.018***
	(1.73)	(2.59)
GrossProfit	0.011***	0.048***
	(3.54)	(6.14)
NetProfit	0.001	-0.035***
	(0.33)	(-3.84)
Liquid	-0.000	-0.003**
	(-0.12)	(-2.25)
Quick	0.001	0.005**
	(0.89)	(2.46)
Cashflow	-0.006***	-0.003
	(-2.98)	(-0.49)
Tangible	-0.003	-0.010
	(-1.39)	(-1.42)
Constant	0.009**	0.017
	(2.10)	(0.62)
Observations	10,728	5,399
Adj.R^2^	0.746	0.907
id FE	YES	YES
Province FE	YES	YES

Robust t-statistics in parentheses

*** p<0.01

** p<0.05

* p<0.1

## 6. Economic consequences

Based on the theoretical analysis and empirical results above, it can be seen that big data tax collection and management has a promoting effect on enterprise innovation. High-quality enterprise development is the target of all enterprises. To further study whether big data tax collection and management can promote enterprise innovation to achieve high-quality enterprise development, measuring the high-quality development of enterprises by the total factor productivity estimated by the LP method [33]. Table 8 presents the results of the economic consequences test. Column (1) shows that Post’s coefficient is 0.433, which is significant at the 1% level. Column (2) shows a coefficient of 0.660 for InnoEff1 and a coefficient of 0.411 for Post, both of which are significant at the 1% level. On the surface, the tax collection and management of big data can promote the high-quality development of enterprises, ensure the continuous development of enterprise innovation, and promote enterprise innovation to better enable the high-quality development of enterprises at a higher level.

**Table 8:** High-quality development of enterprises.

	(1)	(2)
VARIABLES	OLS	Economic consequences
Post	0.433***	0.411***
	(54.55)	(50.74)
InnoEff1		0.660***
		(8.63)
Lev	1.318***	1.308***
	(24.39)	(24.43)
ROA	1.724***	1.746***
	(5.41)	(5.53)
ROE	0.445***	0.435***
	(3.27)	(3.23)
GrossProfit	-0.991***	-1.034***
	(-10.91)	(-11.51)
NetProfit	-0.123	-0.119
	(-1.59)	(-1.53)
Liquid	0.007	0.011
	(0.47)	(0.75)
Quick	-0.000	-0.005
	(-0.01)	(-0.32)
Cashflow	0.804***	0.810***
	(10.70)	(10.88)
Tangible	-0.817***	-0.776***
	(-10.31)	(-9.80)
Constant	10.388***	10.278***
	(37.84)	(37.00)
Observations	16,231	16,204
Adj.R^2^	0.901	0.903
id FE	YES	YES
Province FE	YES	YES

Robust t-statistics in parentheses

*** p<0.01

** p<0.05

* p<0.1

## 7. Discussion

Based on the exogenous impact of China’s "Golden Tax Phase III" pilot project and the research data of A-share listed enterprises from 2010 to 2021, this paper uses the PSM-DID empirical analysis method to analyze the impact of big data tax collection and management on enterprise innovation. This method avoids the problem of policy endogeneity, avoids subjective selective error, and reflects the contribution of each control object to the event, analyzes the impact of the implementation of the policy on cities across the country in other years such as 2013, 2015, and 2016, and comprehensively explains the impact of multi-point implementation of the policy on the event.

In this study, we introduce an innovative method to analyze the impact of big data tax administration on enterprise innovation in further analysis. Different from the traditional methods of existing scholars, who usually use a single dimension to measure corporate innovation, our research defines corporate innovation from two dimensions: development and exploratory innovation, which enriches the methods of measuring corporate innovation when studying the impact of big data tax collection and administration on corporate innovation.

Our analysis captures the changes in the innovation efficiency of enterprises after the "Golden Tax Phase III" pilot, and separates the policy impact of big data tax collection and management on enterprise innovation from other observable factors. The results of the intermediary effect test show that big data tax collection and management can promote enterprise innovation by reducing the financialization of enterprises. The moderating effect test shows that agency cost and financing constraints play a negative moderating role in the impact of big data tax collection and management on enterprise innovation. Heterogeneity analysis shows that the promotion effect of big data tax collection and management on enterprise innovation is more significant in enterprise information transparency and non-high-tech enterprises. Further analysis shows that big data tax collection and management has a more significant role in promoting exploratory innovation in ambidextrous innovation. The results of economic consequence analysis show that big data tax collection and management can promote enterprise innovation and ultimately lead to high-quality development of enterprises.

Based on the above results, this paper draws the following policy implications:

First, improve and make full use of big data analysis technology. The application of digital technologies such as big data and cloud computing in tax collection and administration has greatly improved the supervision of tax departments and played a positive role in enterprise innovation. In the future, the data analysis technology can be further improved and applied to more enterprise non-tax businesses. On the one hand, the government can strengthen the supervision of enterprise behavior and standardize enterprise behavior according to the data analysis results, and on the other hand, it can also use data analysis to track and evaluate the innovation level of enterprises and their industries, and give professional guidance or resource support to enterprise R&D decisions in an appropriate period based on the data analysis results.

Second, build an information sharing mechanism and optimize the market information environment. The digitalization of tax collection and administration has prompted enterprises to improve the quality of information disclosure, which will help enhance investors’ trust in enterprises, thereby improving their risk-taking capabilities. Therefore, government departments can further expand the scope of use of data and information built by the "Golden Tax Phase III" project, build an information sharing mechanism, provide investors with a more accurate reference basis for identifying high-quality enterprises, and help enterprises obtain more resource support when carrying out R&D, mergers and acquisitions and other risk activities.

Third, we should attach importance to the role of innovation and build a barrier against external shocks. The impact of innovation on the development vitality of real enterprises cannot be ignored, when the development of real enterprises is greatly affected by the impact of uncertainty, the government departments should focus on improving the infrastructure environment for the development of real enterprises, guide enterprises to attach importance to innovation, maintain a high degree of attention to the financialization of real enterprises, and work with real enterprises to build an effective barrier against the impact of external uncertainty.

Fourth, improve the internal supervision mechanism of enterprises and standardize the behavior of enterprises. In the institutional environment of strict supervision and transparent information disclosure, only by standardizing enterprise behavior and establishing a good reputation can we survive and develop in a highly competitive market. Therefore, for enterprises, on the one hand, they should continuously improve the internal supervision mechanism, and establish an appropriate management incentive mechanism to encourage the management to increase their willingness to take risks, and strive to improve the future competitiveness and enterprise value of the enterprise. On the other hand, they should take the initiative to improve the quality of information disclosure, standardize their own behavior, and actively use the government’s evaluation mechanism to reduce the information asymmetry between enterprises and investors, win the trust of the market, and improve the efficiency of enterprise innovation.

Overall, the results of the study highlight the need for nuanced policy-making taking into account the implementation of policies at different points in China. Tailored intervention policies are needed to address the unique challenges and opportunities of different companies in terms of innovation.

## 8. Conclusions

The rapid development of information technology such as big data and blockchain has promoted the transformation of tax collection and management in China from "governing taxes by people" to "governing taxes by digits", and improved the ability to supervise tax-related information of enterprises. At the same time, innovation has become the main driving force of national economic growth and an important magic weapon for enterprises to win market competition. As the main body of the national innovation system, real enterprises have become an important factor affecting national and regional economic development.

This study enriches the research on the impact of global tax policy on enterprise innovation, clarifies the difference between big data tax collection and management on the implementation of the "Golden Tax Phase III" policy for enterprise innovation at different points in time, and promotes enterprise innovation by advocating big data tax collection and management, using information technology to establish a sharing mechanism, and improving the supervision efficiency of tax collection and management departments. These insights are of great significance to the international community, provide effective reference for the country’s future tax policy, and play an important role in creating a good business environment, thereby helping to promote the high-quality development of the national economy.

## Supporting information

S1 Table Presents the descriptive statistics of the main variables in this study. (DOCX)

S2 Table Shows the results of substitution of the explanatory variables. (DOCX)
