# Supplementary material for: Big data tax collection and management, enterprise financialization and enterprise innovation: Quasi-natural test evidence based on the "Golden Tax Phase III"
Source: PLoS One. 2024 Dec 19;19(12):e0315222. doi: 10.1371/journal.pone.0315222 (PMC11658519; doi:10.1371/journal.pone.0315222)
Supplement: S1 Table — (DOCX) [file pone.0315222.s001.docx]

**S1 Table.** presents the descriptive statistics of the main variables in this study.

| VarName | Obs | Mean | SD | Min | Median | Max |
| --- | --- | --- | --- | --- | --- | --- |
| Post | 30863 | 0.68 | 0.468 | 0 | 1 | 1 |
| InnoEff1 | 27411 | 0.16 | 0.083 | 0 | 0.1660927 | 0.7294542 |
| InnoEff2 | 27411 | 0.20 | 0.094 | 0 | 0.210732 | 0.8515773 |
| FINRATIO | 30863 | 0.04 | 0.089 | -0.0001789 | 0.0079125 | 0.9721372 |
| Size | 30863 | 22.12 | 1.273 | 19.58496 | 21.92357 | 26.45228 |
| Lev | 30863 | 0.41 | 0.206 | 0.0274426 | 0.3990282 | 0.9078884 |
| ROA | 28626 | 0.04 | 0.065 | -0.3730353 | 0.0395341 | 0.2473081 |
| ROE | 28626 | 0.06 | 0.130 | -0.9257315 | 0.0719132 | 0.4187378 |
| GrossProfit | 30860 | 0.30 | 0.176 | -0.0386154 | 0.2632184 | 0.8712473 |
