# Supplementary material for: Big data tax collection and management, enterprise financialization and enterprise innovation: Quasi-natural test evidence based on the "Golden Tax Phase III"
Source: PLoS One. 2024 Dec 19;19(12):e0315222. doi: 10.1371/journal.pone.0315222 (PMC11658519; doi:10.1371/journal.pone.0315222)
Supplement: S2 Table — (DOCX) [file pone.0315222.s002.docx]

**S2 Table.** shows the results of substitution of the explanatory variables.

|  | (1) | (2) | (3) |
| --- | --- | --- | --- |
| VARIABLES | OLS | FE | FE |
|  |  |  |  |
| Post | 0.032*** | 0.031*** | 0.031*** |
|  | (21.59) | (26.55) | (26.64) |
| Lev | 0.033*** | 0.021*** | 0.021*** |
|  | (6.28) | (3.03) | (3.05) |
| ROA | 0.054 | -0.104*** | -0.102*** |
|  | (1.50) | (-3.28) | (-3.17) |
| ROE | 0.084*** | 0.035** | 0.034** |
|  | (5.34) | (2.57) | (2.51) |
| GrossProfit | -0.025*** | 0.037*** | 0.038*** |
|  | (-4.81) | (3.78) | (3.89) |
| NetProfit | -0.023*** | 0.005 | 0.006 |
|  | (-2.69) | (0.61) | (0.69) |
| Liquid | -0.009*** | 0.000 | 0.000 |
|  | (-5.31) | (0.09) | (0.07) |
| Quick | 0.008*** | -0.000 | -0.000 |
|  | (4.49) | (-0.13) | (-0.12) |
| Cashflow | -0.031** | 0.003 | 0.002 |
|  | (-2.56) | (0.31) | (0.15) |
| Tangible | 0.042*** | -0.065*** | -0.065*** |
|  | (5.26) | (-6.95) | (-6.90) |
| Constant | 0.142*** | 0.205*** | 0.214*** |
|  | (18.33) | (13.60) | (5.93) |
|  |  |  |  |
| Observations | 16,204 | 16,204 | 16,204 |
| Adj.$R^{2}$ | 0.057 | 0.702 | 0.704 |
| id FE |  | YES | YES |
| Province FE |  |  | YES |
